# Age-Friendly University Inventory: Process and Outcome from UMass Lowell Campus

**DOI:** 10.1093/geroni/igaa057.1741

**Published:** 2020-12-16

**Authors:** Ramraj Gautam, Karen Devereaux Melillo, Andrew Hostetler

**Affiliations:** University of Massachusetts Lowell, Lowell, Massachusetts, United States

## Abstract

The UMass Lowell Center for Gerontology Research and Partnerships (CGRP) has been on the Age-Friendly University (AFU) trajectory since April 2018. In April 2019, at the CGRP-sponsored 5th Annual Healthy Aging Living Well Forum, qualitative analysis of table-top discussions identified themes related to the AFU Principles: Accessibility, care, communication community education, flexibility, finances/money, recreation, inclusive society, technology and transportation. In September 2019, in collaboration with the UMass President’s Office and UMass Boston’s Gerontology Institute, we launched the AFU Climate Survey to faculty, staff, and students on the UML campus. This was followed by the AFU Inventory assessments of 12 key campus leaders and offices. Three CGRP members solicited input via email, follow-up telephone communication and information from the University website. The results presented will focus on how other educational institutions might consider proceeding with this process and how to adapt the Inventory to meet other unique campus characteristics.

